# Crystal structures of two isomeric 2-aryl-3-phenyl-1,3-thia­zepan-4-ones

**DOI:** 10.1107/S2056989019010429

**Published:** 2019-07-26

**Authors:** Hemant P. Yennawar, Samuel D. Peterson, Lee J. Silverberg

**Affiliations:** a Pennsylvania State University, Department of Biochemistry and Molecular Biology, 108 Althouse Laboratory, University Park, PA, 16801, USA; b Pennsylvania State University Schuylkill Campus, 200 University Drive, Schuylkill Haven, PA, 17972, USA

**Keywords:** crystal structure, thia­zepan, seven membered ring, chair pucker, isomers

## Abstract

The crystal of 6-(3-nitro­phen­yl)-7-phenyl-5-thia-7-aza­spiro­[2.6]nonan-8-one (**1**), C_19_H_18_N_2_O_3_S, has monoclinic (*P*2_1_/*n*) symmetry while that of its isomer 6-(4-nitro­phen­yl)-7-phenyl-5-thia-7-aza­spiro­[2.6]nonan-8-one (**2**), has ortho­rhom­bic (*Pca*2_1_) symmetry: compound **1** has two mol­ecules, *A* and *B*, in the asymmetric unit while **2** has one. In all three mol­ecules, the seven-membered thia­zepan ring exhibits a chair conformation with *Q*2 and *Q*3 values (Å) of 0.521 (3), 0.735 (3) and 0.485 (3), 0.749 (3) in **1** and 0.517 (5), 0.699 (5) in **2**. In each structure, the phenyl rings attached to adjacent atoms of the thia­zepan ring have inter­planar angles ranging between 41 and 47°. Except for the nitro groups, the three mol­ecules have similar conformations when overlayed in pairs. Both crystal structures are consolidated by C—H⋯O hydrogen bonds.

## Abstract 2

The crystal of 6-(4-nitro­phen­yl)-7-phenyl-5-thia-7-aza­spiro­[2.6]nonan-8-one (**1**), has monoclinic (*P*2_1_/*n*) symmetry while that of its isomer 6-(3-nitro­phen­yl)-7-phenyl-5-thia-7-aza­spiro­[2.6]nonan-8-one (**2**), has ortho­rhom­bic (*Pca*2_1_) symmetry: compound **1** has two mol­ecules, *A* and *B*, in the asymmetric unit while **2** has one. In all three mol­ecules, the seven-membered thia­zepan ring exhibits a chair conformation. Except for the nitro groups, the three mol­ecules have similar conformations when overlayed in pairs.

## Chemical context   

The seven-membered 1,3-thia­zepan-4-one ring system, like the similar six-membered 1,3-thia­zin-4-one and five-membered 1,3-thia­zolidin-4-one systems, is biologically active and of potential medicinal use. For example, the Bristol-Myers Squibb ACE/NEP inhibitor omapatrilat advanced to Phase II clinical trials (Graul *et al.*, 1999 ▸; Robl *et al.* 1997 ▸; Tabrizchi, 2001 ▸; Cozier *et al.* 2018 ▸). In fact, nearly all of the known compounds with this ring system are related in structure to omapatrilat.
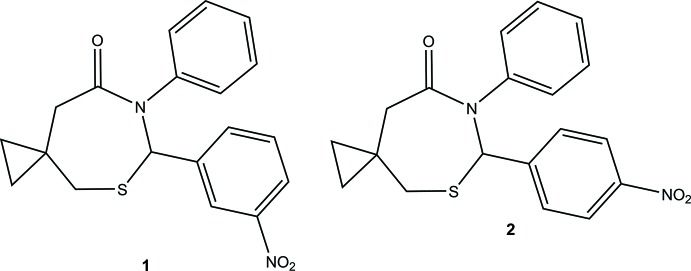


Previously we reported the synthesis and crystal structure of 6,7-diphenyl-5-thia-7-aza­spiro­[2.6]nonan-8-one (Yennawar & Silverberg, 2013 ▸). Herein we report the T3P-promoted synthesis and crystal structures of two new analogs: 6-(4-nitro­phen­yl)-7-phenyl-5-thia-7-aza­spiro­[2.6]nonan-8-one (**1**) and 6-(3-nitro­phen­yl)-7-phenyl-5-thia-7-aza­spiro­[2.6]nonan-8-one (**2**), in which a nitro group substitutes at the *para* and *meta* positions, respectively, of the C-2 aromatic ring.

## Structural commentary   

**Figure 1:**
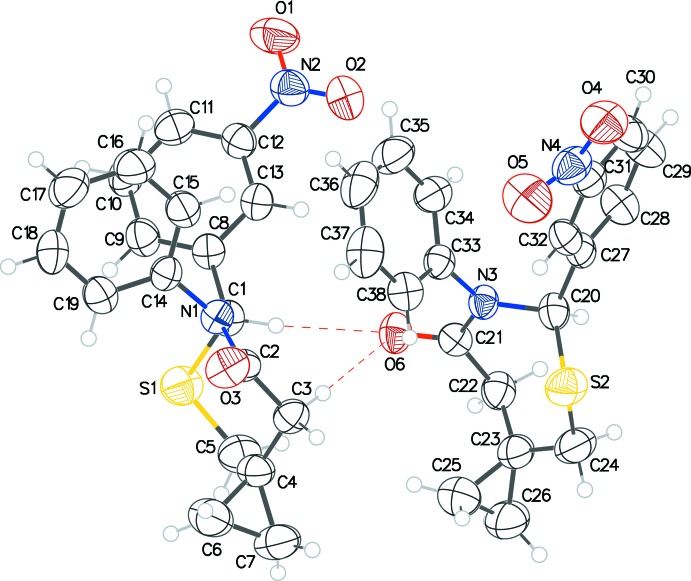
The mol­ecular structure of **1** with displacement ellipsoids drawn at the 50% probability level. C—H⋯O inter­actions are shown as dashed lines.

**Figure 2:**
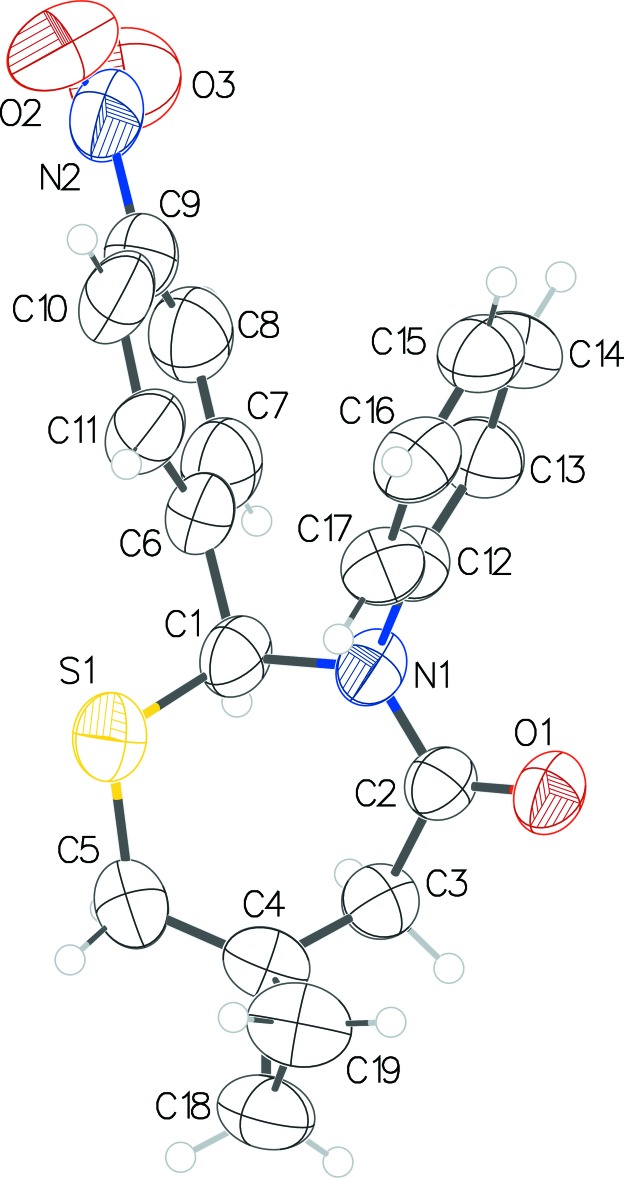
The mol­ecular structure of **2** with displacement ellipsoids drawn at the 50% probability level.

**Figure 3:**
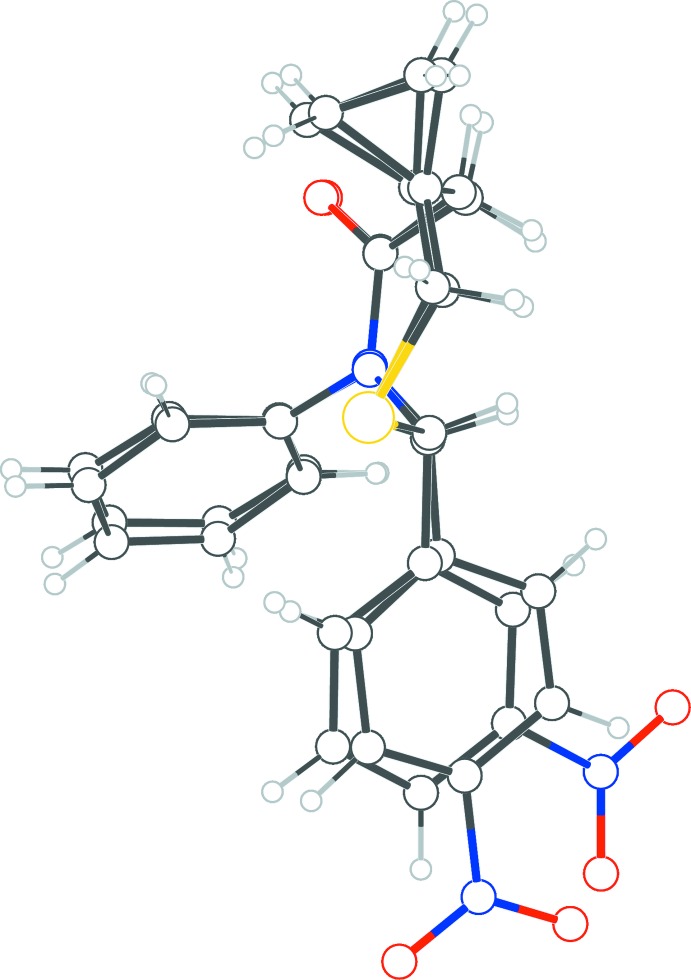
Overlay of the mol­ecule of **2** on the similarly handed mol­ecule of **1**.

Compound **1** crystallizes with two mol­ecules, *A* (containing S1) and *B* (containing S2), in the asymmetric unit (Fig. 1 ▸) and **2** crystallizes with one mol­ecule (Fig. 2 ▸). The configurations of the stereogenic centers in the arbitrarily chosen asymmetric mol­ecules are (*S*) at C1 and (*R*) at C20 for **1** and (*S*) at C1 for **2**; in both structures, crystal symmetry generates a racemic mixture. These mol­ecules adopt similar conformations and overlay closely (Fig. 3 ▸) apart from the nitro groups. The seven-membered thia­zepan rings in both structures adopt chair conformations. The puckering parameters [*Q*2 and *Q*3 (Å)] as calculated by *PLATON* (Spek, 2009 ▸) for mol­ecules *A* and *B* in **1** are 0.521 (3), 0.735 (3) and 0.485 (3), 0.749 (3), respectively, with equivalent values of 0.517 (5), 0.699 (5) for **2**. The dihedral angles between the aromatic rings attached to the 2 and 3 positions of the thia­zepan rings are 46.93 (15) (mol­ecule **1** *A*), 42.50 (15) (**1** *B*) and 42.0 (3)° (**2**).

## Supra­molecular features   

**Figure 4:**
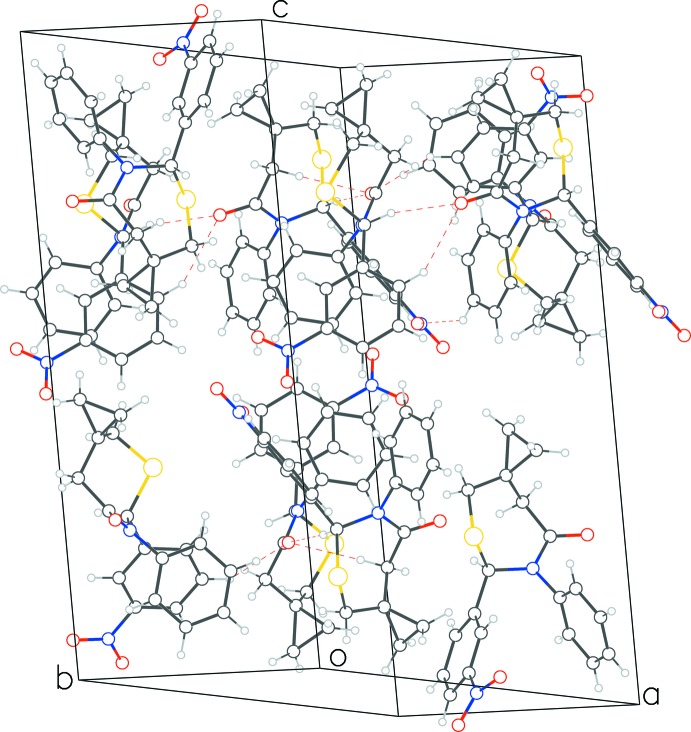
Packing diagram for **1** showing C—H⋯O hydrogen bonds between mol­ecules arranged in the *ab* planes.

**Figure 5:**
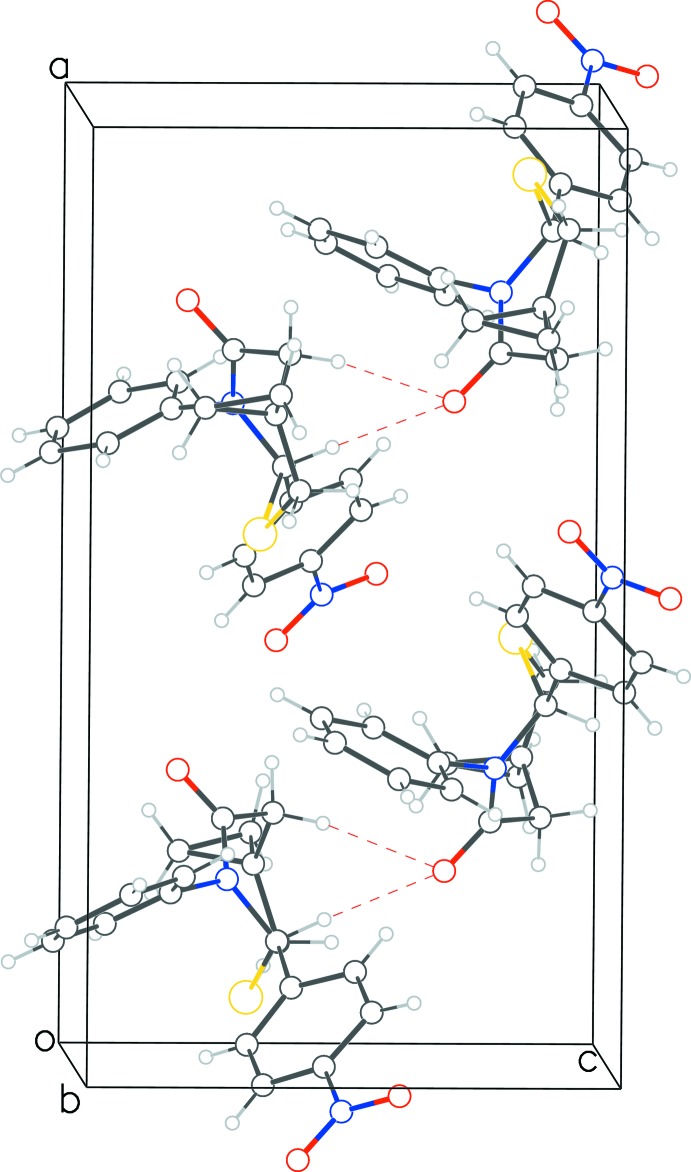
Packing diagram for **2** viewed down the *b*-axis direction.

The extended structure of **1** has more extensive hydrogen bonding compared to that of **2** (Tables 1 ▸ and 2 ▸). In **1**, the mol­ecules are arranged into layers propagating in the *ab* plane, with C—H⋯O hydrogen bonds in both the *a-* and *b*-axis directions, but not in the *c*-axis direction (Fig. 4 ▸). In **2**, the mol­ecules link up *via* C—H⋯O ‘head-to-tail’ hydrogen bonds in the *c*-axis direction (Fig. 5 ▸) and hydro­phobic inter­actions between adjacent chains consolidate the packing in the *a*- and *b*-axis directions.

## Database survey   

A 1,3-thia­zepan-4-one with a 5,6-fused benzene and a 2,3-fused triazole has been reported, but only an *ORTEP* representation was given, without any other data (Bakavoli *et al.*, 2002 ▸). The structures of omapatrilat bound to proteins have been published recently (Cozier, *et al.* 2018 ▸). The 2,3-diphenyl structure that we previously reported showed a chair-type conformation for the thia­zepan ring [CSD (Groom *et al.*, 2016 ▸) refcode MIHVOQ; Yennawar & Silverberg, 2013 ▸] like those reported here.

## Synthesis and crystallization   

A two-necked 25 ml round-bottom flask was oven-dried, cooled under N_2_, and charged with a stir bar. 3- or 4-Nitrobenzaldehyde (0.907 g, 6 mmol), aniline (0.571 g, 6 mmol), and [1-(sulfanylmethyl)cyclopropyl] acetic acid (0.877 g, 6 mmol) were added. Pyridine (1.95 ml, 24 mmol) was added. Finally, 2,4,6-tripropyl-1,3,5,2,4,6-trioxatri­phospho­rinane-2,4,6-tri­oxide (T3P) in 2-methyl­tetra­hydro­furan (50 weight %; 7.3 ml, 12 mmol) was added. The reaction was stirred at room temperature and followed by TLC. The mixture was poured into a separatory funnel with di­chloro­methane and distilled water. The layers were separated and the aqueous layer was then extracted twice with di­chloro­methane. The organic fractions were combined and washed with saturated sodium bicarbonate and saturated sodium chloride and then dried over sodium sulfate and concentrated under vacuum. Further purification was carried out as indicated below for each compound.

6-(3-Nitro­phen­yl)-7-phenyl-5-thia-7-aza­spiro­[2.6]nonan-8-one (**1**): Chromatography on 30 g flash silica gel with mixtures of ethyl acetate and hexa­nes gave a solid. Recrystallization from 2-propanol solution gave crystals (0.5192 g, 26%), m.p. 457–458 K. X-ray-quality crystals were grown by slow evaporation from a 2-propanol solution.

6-(4-Nitro­phen­yl)-7-phenyl-5-thia-7-aza­spiro­[2.6]nonan-8-one (**2**): Chromatography on 30 g flash silica gel with mixtures of ethyl acetate and hexa­nes gave a solid. Recrystallization from ethyl acetate solution gave colorless crystals (0.1804 g, 9%), m.p. 480–482 K (decomposition). X-ray-quality crystals were grown by slow evaporation from an ethyl acetate solution.

## Refinement   

Crystal data, data collection and structure refinement details are summarized in Table 3 ▸. The hydrogen atoms were placed geometrically (C—H = 0.93–0.98 Å) and refined as riding on their parent atoms with *U*_iso_(H) = 1.2*U*_eq_(C).

## Supplementary Material

Crystal structure: contains datablock(s) 1, 2. DOI: 10.1107/S2056989019010429/hb7839sup1.cif

Structure factors: contains datablock(s) 1. DOI: 10.1107/S2056989019010429/hb78391sup2.hkl

Supporting information file. DOI: 10.1107/S2056989019010429/hb78391sup5.mol

Structure factors: contains datablock(s) 2. DOI: 10.1107/S2056989019010429/hb78392sup4.hkl

Supporting information file. DOI: 10.1107/S2056989019010429/hb78392sup6.mol

Supporting information file. DOI: 10.1107/S2056989019010429/hb78391sup6.cml

Supporting information file. DOI: 10.1107/S2056989019010429/hb78392sup7.cml

CCDC references: 1942358, 1942357

Additional supporting information:  crystallographic information; 3D view; checkCIF report

## Figures and Tables

**Table 1:** Hydrogen-bond geometry (Å, °) for **1** [Chem scheme1]

*D*—H⋯*A*	*D*—H	H⋯*A*	*D*⋯*A*	*D*—H⋯*A*
C1—H1⋯O6	0.98	2.41	3.390 (3)	175
C3—H3*B*⋯O6	0.97	2.50	3.469 (3)	174
C10—H10⋯O6^i^	0.93	2.60	3.408 (3)	146
C17—H17⋯O2^ii^	0.93	2.46	3.216 (4)	138
C20—H20⋯O3^iii^	0.98	2.41	3.369 (3)	165
C28—H28⋯O3^iii^	0.93	2.56	3.384 (4)	147

Symmetry codes: (i) 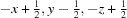; (ii) 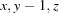; (iii) 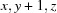.

**Table 2:** Hydrogen-bond geometry (Å, °) for **2** [Chem scheme1]

*D*—H⋯*A*	*D*—H	H⋯*A*	*D*⋯*A*	*D*—H⋯*A*
C1—H1⋯O1^i^	0.98	2.38	3.352 (6)	173
C3—H3*B*⋯O1^i^	0.97	2.48	3.444 (5)	171

Symmetry code: (i) 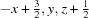.

**Table 3:** Experimental details

	**1**	**2**
Crystal data		
Chemical formula	C_19_H_18_N_2_O_3_S	C_19_H_18_N_2_O_3_S
*M*_r_	354.41	354.41
Crystal system, space group	Monoclinic, *P*2_1_/*n*	Orthorhombic, *P* *c* *a*2_1_
Temperature (K)	298	298
*a*, *b*, *c* (Å)	16.993 (4), 9.955 (2), 21.243 (5)	17.478 (3), 10.4125 (19), 9.7129 (17)
α, β, γ (°)	90, 99.531 (4), 90	90, 90, 90
*V* (Å^3^)	3543.9 (15)	1767.6 (5)
*Z*	8	4
Radiation type	Mo *K*α	Mo *K*α
μ (mm^−1^)	0.20	0.20
Crystal size (mm)	0.24 × 0.12 × 0.09	0.27 × 0.1 × 0.04

Data collection		
Diffractometer	Bruker SMART CCD area detector	Bruker SMART CCD area detector
Absorption correction	Multi-scan (*SADABS*; Bruker, 2001 ▸)	Multi-scan (*SADABS*; Bruker, 2001 ▸)
*T*_min_, *T*_max_	0.780, 0.9	0.769, 0.9
No. of measured, independent and observed [*I* > 2σ(*I*)] reflections	30602, 8495, 3560	15240, 4261, 2403
*R*_int_	0.083	0.056
(sin θ/λ)_max_ (Å^−1^)	0.669	0.668

Refinement		
*R*[*F* ^2^ > 2σ(*F* ^2^)], *wR*(*F* ^2^), *S*	0.057, 0.155, 0.94	0.081, 0.249, 1.01
No. of reflections	8495	4261
No. of parameters	451	226
No. of restraints	0	1
H-atom treatment	H-atom parameters constrained	H-atom parameters constrained
Δρ_max_, Δρ_min_ (e Å^−3^)	0.25, −0.26	0.55, −0.30
Absolute structure	–	Flack (1983 ▸)
Absolute structure parameter	–	0.47 (19)

Computer programs: *SMART* and *SAINT* (Bruker, 2001 ▸), *SHELXS* (Sheldrick, 2008 ▸), *SHELXL* (Sheldrick, 2015 ▸) and *OLEX2* (Dolomanov *et al.*, 2009 ▸).

## supplementary crystallographic information

### 6-(3-Nitrophenyl)-7-phenyl-5-thia-7-azaspiro[2.6]nonan-8-one (1) Crystal data

**Table:**

C_19_H_18_N_2_O_3_S	*F*(000) = 1488
*M**_r_* = 354.41	*D*_x_ = 1.329 Mg m^−^^3^
Monoclinic, *P*2_1_/*n*	Mo *K*α radiation, λ = 0.71073 Å
*a* = 16.993 (4) Å	Cell parameters from 2681 reflections
*b* = 9.955 (2) Å	θ = 2.4–28.3°
*c* = 21.243 (5) Å	µ = 0.20 mm^−^^1^
β = 99.531 (4)°	*T* = 298 K
*V* = 3543.9 (15) Å^3^	Block, colorless
*Z* = 8	0.24 × 0.12 × 0.09 mm

### 6-(3-Nitrophenyl)-7-phenyl-5-thia-7-azaspiro[2.6]nonan-8-one (1) Data collection

**Table:**

Bruker SMART CCD area detector diffractometer	8495 independent reflections
Radiation source: fine-focus sealed tube	3560 reflections with *I* > 2σ(*I*)
Parallel-graphite monochromator	*R*_int_ = 0.083
Detector resolution: 8.34 pixels mm^-1^	θ_max_ = 28.4°, θ_min_ = 1.4°
phi and ω scans	*h* = −21→22
Absorption correction: multi-scan (SADABS; Bruker, 2001)	*k* = −12→13
*T*_min_ = 0.780, *T*_max_ = 0.9	*l* = −26→28
30602 measured reflections	

### 6-(3-Nitrophenyl)-7-phenyl-5-thia-7-azaspiro[2.6]nonan-8-one (1) Refinement

**Table:**

Refinement on *F*^2^	Primary atom site location: structure-invariant direct methods
Least-squares matrix: full	Secondary atom site location: difference Fourier map
*R*[*F*^2^ > 2σ(*F*^2^)] = 0.057	Hydrogen site location: inferred from neighbouring sites
*wR*(*F*^2^) = 0.155	H-atom parameters constrained
*S* = 0.94	*w* = 1/[σ^2^(*F*_o_^2^) + (0.0603*P*)^2^] where *P* = (*F*_o_^2^ + 2*F*_c_^2^)/3
8495 reflections	(Δ/σ)_max_ < 0.001
451 parameters	Δρ_max_ = 0.25 e Å^−^^3^
0 restraints	Δρ_min_ = −0.26 e Å^−^^3^

### 6-(3-Nitrophenyl)-7-phenyl-5-thia-7-azaspiro[2.6]nonan-8-one (1) Special details

**Table:**

Experimental. The data collection nominally covered a full sphere of reciprocal space by a combination of 4 sets of ω scans each set at different φ and/or 2θ angles and each scan (10 s exposure) covering -0.300° degrees in ω. The crystal to detector distance was 5.86 cm.
Geometry. All esds (except the esd in the dihedral angle between two l.s. planes) are estimated using the full covariance matrix. The cell esds are taken into account individually in the estimation of esds in distances, angles and torsion angles; correlations between esds in cell parameters are only used when they are defined by crystal symmetry. An approximate (isotropic) treatment of cell esds is used for estimating esds involving l.s. planes.
Refinement. Refinement of F^2^ against ALL reflections. The weighted R-factor wR and goodness of fit S are based on F^2^, conventional R-factors R are based on F, with F set to zero for negative F^2^. The threshold expression of F^2^ > 2sigma(F^2^) is used only for calculating R-factors(gt) etc. and is not relevant to the choice of reflections for refinement. R-factors based on F^2^ are statistically about twice as large as those based on F, and R- factors based on ALL data will be even larger.

### 6-(3-Nitrophenyl)-7-phenyl-5-thia-7-azaspiro[2.6]nonan-8-one (1) Fractional atomic coordinates and isotropic or equivalent isotropic displacement parameters (Å^2^)

**Table:**

	*x*	*y*	*z*	*U*_iso_*/*U*_eq_	
C1	0.38777 (15)	0.3850 (3)	0.24330 (13)	0.0472 (7)	
H1	0.4111	0.4733	0.2374	0.057*	
C2	0.52215 (16)	0.2834 (3)	0.24439 (13)	0.0458 (7)	
C3	0.53440 (16)	0.3814 (3)	0.19259 (13)	0.0497 (7)	
H3A	0.5906	0.3831	0.1890	0.060*	
H3B	0.5197	0.4707	0.2048	0.060*	
C4	0.48636 (17)	0.3471 (3)	0.12818 (13)	0.0529 (7)	
C5	0.40156 (18)	0.3958 (3)	0.11569 (14)	0.0673 (9)	
H5A	0.3793	0.3748	0.0718	0.081*	
H5B	0.4021	0.4928	0.1198	0.081*	
C6	0.5063 (2)	0.2202 (3)	0.09693 (16)	0.0785 (10)	
H6A	0.4628	0.1711	0.0717	0.094*	
H6B	0.5485	0.1646	0.1197	0.094*	
C7	0.5293 (2)	0.3518 (3)	0.07165 (15)	0.0748 (10)	
H7A	0.4996	0.3826	0.0313	0.090*	
H7B	0.5853	0.3761	0.0793	0.090*	
C8	0.32870 (15)	0.4017 (3)	0.28901 (13)	0.0461 (7)	
C9	0.26482 (16)	0.3140 (3)	0.28908 (14)	0.0551 (8)	
H9	0.2578	0.2431	0.2602	0.066*	
C10	0.21165 (16)	0.3302 (3)	0.33116 (15)	0.0598 (8)	
H10	0.1691	0.2710	0.3301	0.072*	
C11	0.22164 (17)	0.4337 (3)	0.37465 (15)	0.0582 (8)	
H11	0.1861	0.4458	0.4031	0.070*	
C12	0.28542 (16)	0.5189 (3)	0.37502 (13)	0.0496 (7)	
C13	0.33890 (16)	0.5053 (3)	0.33310 (13)	0.0484 (7)	
H13	0.3813	0.5649	0.3345	0.058*	
C14	0.43844 (15)	0.1901 (3)	0.31425 (14)	0.0456 (7)	
C15	0.45269 (18)	0.2164 (3)	0.37871 (15)	0.0593 (8)	
H15	0.4730	0.2995	0.3935	0.071*	
C16	0.4370 (2)	0.1200 (4)	0.42134 (16)	0.0750 (10)	
H16	0.4467	0.1380	0.4649	0.090*	
C17	0.4070 (2)	−0.0028 (3)	0.39960 (18)	0.0771 (10)	
H17	0.3961	−0.0679	0.4283	0.093*	
C18	0.39337 (19)	−0.0286 (3)	0.33550 (18)	0.0715 (9)	
H18	0.3733	−0.1118	0.3209	0.086*	
C19	0.40877 (17)	0.0665 (3)	0.29254 (15)	0.0582 (8)	
H19	0.3993	0.0479	0.2490	0.070*	
N1	0.45322 (12)	0.2936 (2)	0.27006 (10)	0.0443 (6)	
N2	0.29752 (18)	0.6265 (3)	0.42288 (12)	0.0603 (7)	
O1	0.24710 (15)	0.6440 (2)	0.45664 (11)	0.0846 (7)	
O2	0.35847 (16)	0.6932 (2)	0.42706 (11)	0.0858 (7)	
O3	0.57139 (11)	0.19619 (19)	0.26254 (10)	0.0632 (6)	
S1	0.33553 (5)	0.32922 (9)	0.16613 (4)	0.0704 (3)	
C20	0.64468 (15)	0.8845 (3)	0.29410 (13)	0.0484 (7)	
H20	0.6223	0.9700	0.2766	0.058*	
C21	0.52037 (16)	0.7803 (3)	0.23659 (13)	0.0435 (7)	
C22	0.52413 (16)	0.8762 (3)	0.18223 (13)	0.0538 (8)	
H22A	0.5306	0.9668	0.1992	0.065*	
H22B	0.4738	0.8727	0.1531	0.065*	
C23	0.59097 (18)	0.8473 (3)	0.14525 (14)	0.0594 (8)	
C24	0.67190 (18)	0.9037 (4)	0.17153 (14)	0.0725 (10)	
H24A	0.6666	0.9999	0.1766	0.087*	
H24B	0.7066	0.8894	0.1402	0.087*	
C25	0.5874 (2)	0.7178 (4)	0.10892 (16)	0.0812 (10)	
H25A	0.6374	0.6731	0.1062	0.097*	
H25B	0.5429	0.6581	0.1114	0.097*	
C26	0.5701 (2)	0.8472 (4)	0.07334 (16)	0.0862 (11)	
H26A	0.5152	0.8656	0.0545	0.103*	
H26B	0.6097	0.8807	0.0492	0.103*	
C27	0.68160 (15)	0.9101 (3)	0.36255 (13)	0.0473 (7)	
C28	0.66026 (17)	1.0246 (3)	0.39258 (15)	0.0611 (8)	
H28	0.6247	1.0854	0.3700	0.073*	
C29	0.6914 (2)	1.0492 (4)	0.45580 (16)	0.0747 (10)	
H29	0.6770	1.1271	0.4752	0.090*	
C30	0.74311 (19)	0.9604 (4)	0.49030 (15)	0.0686 (9)	
H30	0.7634	0.9761	0.5331	0.082*	
C31	0.76402 (16)	0.8479 (3)	0.45990 (14)	0.0525 (7)	
C32	0.73499 (16)	0.8210 (3)	0.39678 (14)	0.0514 (7)	
H32	0.7510	0.7442	0.3774	0.062*	
C33	0.58190 (15)	0.6820 (3)	0.33613 (13)	0.0439 (7)	
C34	0.55008 (18)	0.7007 (3)	0.39071 (15)	0.0611 (8)	
H34	0.5234	0.7801	0.3966	0.073*	
C35	0.5576 (2)	0.6016 (4)	0.43721 (16)	0.0797 (10)	
H35	0.5369	0.6149	0.4746	0.096*	
C36	0.5957 (2)	0.4837 (4)	0.42757 (18)	0.0775 (11)	
H36	0.6007	0.4169	0.4586	0.093*	
C37	0.62626 (18)	0.4640 (3)	0.37303 (19)	0.0719 (10)	
H37	0.6514	0.3832	0.3667	0.086*	
C38	0.62024 (16)	0.5631 (3)	0.32684 (14)	0.0549 (8)	
H38	0.6418	0.5497	0.2898	0.066*	
N3	0.57806 (12)	0.7883 (2)	0.28935 (10)	0.0425 (5)	
N4	0.81967 (15)	0.7509 (3)	0.49575 (14)	0.0637 (7)	
O4	0.85870 (14)	0.7871 (2)	0.54671 (11)	0.0852 (7)	
O5	0.82594 (15)	0.6398 (3)	0.47322 (12)	0.0904 (8)	
O6	0.46794 (11)	0.69436 (18)	0.23280 (9)	0.0551 (5)	
S2	0.72025 (4)	0.83452 (9)	0.24715 (4)	0.0681 (3)	

### 6-(3-Nitrophenyl)-7-phenyl-5-thia-7-azaspiro[2.6]nonan-8-one (1) Atomic displacement parameters (Å^2^)

**Table:**

	*U*^11^	*U*^22^	*U*^33^	*U*^12^	*U*^13^	*U*^23^
C1	0.0411 (15)	0.0448 (17)	0.0557 (18)	0.0023 (13)	0.0080 (13)	0.0011 (14)
C2	0.0440 (16)	0.0494 (18)	0.0444 (17)	0.0019 (14)	0.0080 (13)	−0.0006 (14)
C3	0.0465 (16)	0.0500 (17)	0.0548 (19)	0.0047 (13)	0.0146 (14)	0.0025 (15)
C4	0.0653 (19)	0.0506 (18)	0.0436 (18)	0.0059 (15)	0.0115 (15)	0.0017 (15)
C5	0.070 (2)	0.077 (2)	0.053 (2)	0.0036 (18)	0.0039 (16)	0.0057 (17)
C6	0.110 (3)	0.066 (2)	0.060 (2)	0.008 (2)	0.016 (2)	−0.0039 (19)
C7	0.093 (2)	0.079 (3)	0.058 (2)	0.010 (2)	0.0283 (19)	0.0086 (19)
C8	0.0420 (15)	0.0431 (17)	0.0531 (18)	0.0012 (13)	0.0072 (14)	−0.0008 (14)
C9	0.0480 (16)	0.0530 (19)	0.064 (2)	−0.0036 (14)	0.0083 (15)	−0.0085 (15)
C10	0.0428 (16)	0.065 (2)	0.073 (2)	−0.0086 (15)	0.0132 (16)	−0.0031 (18)
C11	0.0503 (18)	0.067 (2)	0.059 (2)	0.0045 (16)	0.0153 (15)	0.0025 (18)
C12	0.0555 (18)	0.0419 (17)	0.0504 (19)	0.0056 (14)	0.0064 (15)	0.0006 (15)
C13	0.0439 (15)	0.0446 (17)	0.0564 (19)	−0.0010 (13)	0.0072 (14)	0.0023 (15)
C14	0.0462 (15)	0.0408 (17)	0.0502 (19)	−0.0002 (13)	0.0090 (13)	0.0037 (15)
C15	0.074 (2)	0.0500 (19)	0.053 (2)	−0.0070 (16)	0.0077 (16)	−0.0028 (16)
C16	0.112 (3)	0.064 (2)	0.052 (2)	−0.004 (2)	0.020 (2)	0.0035 (19)
C17	0.102 (3)	0.059 (2)	0.077 (3)	−0.001 (2)	0.035 (2)	0.013 (2)
C18	0.086 (2)	0.046 (2)	0.084 (3)	−0.0154 (17)	0.020 (2)	0.001 (2)
C19	0.067 (2)	0.0486 (19)	0.059 (2)	−0.0024 (16)	0.0108 (16)	−0.0057 (17)
N1	0.0422 (13)	0.0429 (13)	0.0483 (14)	0.0020 (10)	0.0087 (11)	0.0027 (11)
N2	0.0754 (19)	0.0507 (17)	0.0552 (18)	0.0073 (15)	0.0121 (15)	0.0002 (14)
O1	0.1002 (18)	0.0907 (18)	0.0683 (16)	0.0102 (15)	0.0300 (14)	−0.0169 (14)
O2	0.1053 (19)	0.0678 (16)	0.0867 (18)	−0.0234 (14)	0.0228 (15)	−0.0174 (13)
O3	0.0565 (12)	0.0635 (14)	0.0708 (14)	0.0210 (11)	0.0139 (11)	0.0136 (11)
S1	0.0524 (5)	0.0998 (7)	0.0557 (5)	−0.0098 (4)	−0.0008 (4)	−0.0024 (5)
C20	0.0453 (15)	0.0448 (17)	0.0525 (18)	−0.0066 (13)	0.0007 (14)	0.0001 (14)
C21	0.0384 (15)	0.0459 (17)	0.0455 (17)	0.0038 (13)	0.0051 (13)	−0.0022 (14)
C22	0.0504 (17)	0.0551 (19)	0.0525 (19)	−0.0061 (14)	−0.0017 (14)	0.0043 (15)
C23	0.0622 (19)	0.070 (2)	0.0460 (19)	−0.0133 (17)	0.0093 (15)	0.0019 (16)
C24	0.069 (2)	0.099 (3)	0.051 (2)	−0.0195 (19)	0.0147 (17)	0.0054 (19)
C25	0.086 (3)	0.085 (3)	0.076 (2)	−0.008 (2)	0.024 (2)	−0.015 (2)
C26	0.092 (3)	0.113 (3)	0.054 (2)	−0.027 (2)	0.0099 (19)	0.003 (2)
C27	0.0440 (15)	0.0468 (17)	0.0491 (18)	−0.0076 (14)	0.0019 (14)	−0.0021 (15)
C28	0.0618 (19)	0.056 (2)	0.063 (2)	0.0039 (16)	0.0020 (16)	−0.0065 (17)
C29	0.082 (2)	0.071 (2)	0.069 (2)	0.0066 (19)	0.006 (2)	−0.023 (2)
C30	0.066 (2)	0.084 (3)	0.053 (2)	−0.0101 (19)	0.0008 (17)	−0.013 (2)
C31	0.0452 (16)	0.059 (2)	0.0519 (19)	−0.0063 (15)	0.0033 (14)	0.0022 (17)
C32	0.0483 (16)	0.0521 (18)	0.0513 (19)	−0.0059 (14)	0.0013 (14)	−0.0077 (15)
C33	0.0386 (14)	0.0460 (17)	0.0453 (17)	−0.0038 (13)	0.0017 (13)	0.0016 (14)
C34	0.066 (2)	0.062 (2)	0.059 (2)	−0.0022 (16)	0.0184 (17)	0.0022 (18)
C35	0.092 (3)	0.093 (3)	0.056 (2)	−0.019 (2)	0.017 (2)	0.013 (2)
C36	0.075 (2)	0.080 (3)	0.070 (3)	−0.020 (2)	−0.012 (2)	0.031 (2)
C37	0.058 (2)	0.056 (2)	0.096 (3)	0.0022 (16)	−0.007 (2)	0.016 (2)
C38	0.0510 (17)	0.0523 (19)	0.060 (2)	0.0010 (15)	0.0058 (15)	0.0003 (17)
N3	0.0404 (12)	0.0415 (13)	0.0435 (14)	−0.0048 (10)	0.0007 (11)	0.0036 (11)
N4	0.0548 (16)	0.080 (2)	0.0546 (19)	−0.0085 (16)	0.0048 (14)	0.0122 (17)
O4	0.0749 (15)	0.117 (2)	0.0558 (15)	−0.0125 (14)	−0.0133 (13)	0.0113 (14)
O5	0.1009 (19)	0.0781 (18)	0.0858 (19)	0.0195 (15)	−0.0034 (15)	0.0073 (15)
O6	0.0462 (11)	0.0529 (12)	0.0628 (13)	−0.0086 (10)	−0.0012 (10)	0.0042 (10)
S2	0.0479 (4)	0.0946 (7)	0.0624 (6)	−0.0072 (4)	0.0112 (4)	−0.0025 (5)

### 6-(3-Nitrophenyl)-7-phenyl-5-thia-7-azaspiro[2.6]nonan-8-one (1) Geometric parameters (Å, º)

**Table:**

C1—H1	0.9800	C20—H20	0.9800
C1—C8	1.517 (4)	C20—C27	1.507 (4)
C1—N1	1.476 (3)	C20—N3	1.474 (3)
C1—S1	1.817 (3)	C20—S2	1.821 (3)
C2—C3	1.510 (4)	C21—C22	1.508 (4)
C2—N1	1.375 (3)	C21—N3	1.364 (3)
C2—O3	1.223 (3)	C21—O6	1.228 (3)
C3—H3A	0.9700	C22—H22A	0.9700
C3—H3B	0.9700	C22—H22B	0.9700
C3—C4	1.512 (4)	C22—C23	1.512 (4)
C4—C5	1.502 (4)	C23—C24	1.505 (4)
C4—C6	1.491 (4)	C23—C25	1.499 (4)
C4—C7	1.505 (4)	C23—C26	1.510 (4)
C5—H5A	0.9700	C24—H24A	0.9700
C5—H5B	0.9700	C24—H24B	0.9700
C5—S1	1.801 (3)	C24—S2	1.814 (3)
C6—H6A	0.9700	C25—H25A	0.9700
C6—H6B	0.9700	C25—H25B	0.9700
C6—C7	1.492 (4)	C25—C26	1.498 (5)
C7—H7A	0.9700	C26—H26A	0.9700
C7—H7B	0.9700	C26—H26B	0.9700
C8—C9	1.393 (4)	C27—C28	1.384 (4)
C8—C13	1.384 (4)	C27—C32	1.385 (4)
C9—H9	0.9300	C28—H28	0.9300
C9—C10	1.382 (4)	C28—C29	1.381 (4)
C10—H10	0.9300	C29—H29	0.9300
C10—C11	1.376 (4)	C29—C30	1.370 (4)
C11—H11	0.9300	C30—H30	0.9300
C11—C12	1.375 (4)	C30—C31	1.369 (4)
C12—C13	1.380 (4)	C31—C32	1.376 (4)
C12—N2	1.468 (4)	C31—N4	1.472 (4)
C13—H13	0.9300	C32—H32	0.9300
C14—C15	1.376 (4)	C33—C34	1.371 (4)
C14—C19	1.379 (4)	C33—C38	1.381 (4)
C14—N1	1.444 (3)	C33—N3	1.446 (3)
C15—H15	0.9300	C34—H34	0.9300
C15—C16	1.375 (4)	C34—C35	1.387 (4)
C16—H16	0.9300	C35—H35	0.9300
C16—C17	1.374 (4)	C35—C36	1.372 (5)
C17—H17	0.9300	C36—H36	0.9300
C17—C18	1.367 (4)	C36—C37	1.360 (5)
C18—H18	0.9300	C37—H37	0.9300
C18—C19	1.370 (4)	C37—C38	1.383 (4)
C19—H19	0.9300	C38—H38	0.9300
N2—O1	1.217 (3)	N4—O4	1.226 (3)
N2—O2	1.221 (3)	N4—O5	1.216 (3)
			
C8—C1—H1	107.9	C27—C20—H20	107.1
C8—C1—S1	109.56 (18)	C27—C20—S2	110.86 (18)
N1—C1—H1	107.9	N3—C20—H20	107.1
N1—C1—C8	110.9 (2)	N3—C20—C27	111.6 (2)
N1—C1—S1	112.55 (18)	N3—C20—S2	112.84 (18)
S1—C1—H1	107.9	S2—C20—H20	107.1
N1—C2—C3	118.1 (2)	N3—C21—C22	118.5 (2)
O3—C2—C3	121.2 (3)	O6—C21—C22	120.9 (2)
O3—C2—N1	120.7 (3)	O6—C21—N3	120.6 (2)
C2—C3—H3A	108.9	C21—C22—H22A	108.7
C2—C3—H3B	108.9	C21—C22—H22B	108.7
C2—C3—C4	113.2 (2)	C21—C22—C23	114.1 (2)
H3A—C3—H3B	107.7	H22A—C22—H22B	107.6
C4—C3—H3A	108.9	C23—C22—H22A	108.7
C4—C3—H3B	108.9	C23—C22—H22B	108.7
C5—C4—C3	116.4 (2)	C22—C23—C26	117.1 (3)
C6—C4—C3	117.8 (3)	C24—C23—C22	117.1 (3)
C6—C4—C5	118.5 (3)	C24—C23—C26	115.0 (3)
C6—C4—C7	59.7 (2)	C25—C23—C22	117.5 (3)
C7—C4—C3	117.0 (3)	C25—C23—C24	117.9 (3)
C7—C4—C5	115.2 (3)	C25—C23—C26	59.7 (2)
C4—C5—H5A	108.1	C23—C24—H24A	108.3
C4—C5—H5B	108.1	C23—C24—H24B	108.3
C4—C5—S1	116.8 (2)	C23—C24—S2	116.0 (2)
H5A—C5—H5B	107.3	H24A—C24—H24B	107.4
S1—C5—H5A	108.1	S2—C24—H24A	108.3
S1—C5—H5B	108.1	S2—C24—H24B	108.3
C4—C6—H6A	117.7	C23—C25—H25A	117.7
C4—C6—H6B	117.7	C23—C25—H25B	117.7
C4—C6—C7	60.6 (2)	H25A—C25—H25B	114.8
H6A—C6—H6B	114.8	C26—C25—C23	60.5 (2)
C7—C6—H6A	117.7	C26—C25—H25A	117.7
C7—C6—H6B	117.7	C26—C25—H25B	117.7
C4—C7—H7A	117.8	C23—C26—H26A	117.8
C4—C7—H7B	117.8	C23—C26—H26B	117.8
C6—C7—C4	59.7 (2)	C25—C26—C23	59.8 (2)
C6—C7—H7A	117.8	C25—C26—H26A	117.8
C6—C7—H7B	117.8	C25—C26—H26B	117.8
H7A—C7—H7B	114.9	H26A—C26—H26B	114.9
C9—C8—C1	122.0 (3)	C28—C27—C20	119.0 (3)
C13—C8—C1	119.5 (2)	C28—C27—C32	119.0 (3)
C13—C8—C9	118.4 (3)	C32—C27—C20	122.0 (3)
C8—C9—H9	119.3	C27—C28—H28	119.8
C10—C9—C8	121.4 (3)	C29—C28—C27	120.4 (3)
C10—C9—H9	119.3	C29—C28—H28	119.8
C9—C10—H10	120.0	C28—C29—H29	119.5
C11—C10—C9	120.0 (3)	C30—C29—C28	121.0 (3)
C11—C10—H10	120.0	C30—C29—H29	119.5
C10—C11—H11	120.8	C29—C30—H30	121.0
C10—C11—C12	118.4 (3)	C29—C30—C31	118.0 (3)
C12—C11—H11	120.8	C31—C30—H30	121.0
C11—C12—C13	122.6 (3)	C30—C31—C32	122.7 (3)
C11—C12—N2	118.3 (3)	C30—C31—N4	119.2 (3)
C13—C12—N2	119.1 (3)	C32—C31—N4	118.2 (3)
C8—C13—H13	120.4	C27—C32—H32	120.5
C12—C13—C8	119.2 (3)	C31—C32—C27	119.0 (3)
C12—C13—H13	120.4	C31—C32—H32	120.5
C15—C14—C19	119.8 (3)	C34—C33—C38	119.9 (3)
C15—C14—N1	119.3 (2)	C34—C33—N3	120.2 (3)
C19—C14—N1	120.8 (3)	C38—C33—N3	119.9 (3)
C14—C15—H15	120.0	C33—C34—H34	119.9
C16—C15—C14	120.1 (3)	C33—C34—C35	120.2 (3)
C16—C15—H15	120.0	C35—C34—H34	119.9
C15—C16—H16	120.0	C34—C35—H35	120.3
C17—C16—C15	120.0 (3)	C36—C35—C34	119.5 (3)
C17—C16—H16	120.0	C36—C35—H35	120.3
C16—C17—H17	120.2	C35—C36—H36	119.8
C18—C17—C16	119.6 (3)	C37—C36—C35	120.5 (3)
C18—C17—H17	120.2	C37—C36—H36	119.8
C17—C18—H18	119.5	C36—C37—H37	119.7
C17—C18—C19	120.9 (3)	C36—C37—C38	120.5 (3)
C19—C18—H18	119.5	C38—C37—H37	119.7
C14—C19—H19	120.2	C33—C38—C37	119.4 (3)
C18—C19—C14	119.5 (3)	C33—C38—H38	120.3
C18—C19—H19	120.2	C37—C38—H38	120.3
C2—N1—C1	122.0 (2)	C21—N3—C20	122.3 (2)
C2—N1—C14	117.2 (2)	C21—N3—C33	117.8 (2)
C14—N1—C1	119.3 (2)	C33—N3—C20	118.8 (2)
O1—N2—C12	118.7 (3)	O4—N4—C31	118.1 (3)
O1—N2—O2	123.4 (3)	O5—N4—C31	118.9 (3)
O2—N2—C12	117.8 (3)	O5—N4—O4	123.0 (3)
C5—S1—C1	99.49 (14)	C24—S2—C20	97.13 (14)
			
C1—C8—C9—C10	179.8 (3)	C20—C27—C28—C29	178.3 (3)
C1—C8—C13—C12	−179.3 (2)	C20—C27—C32—C31	−177.4 (2)
C2—C3—C4—C5	84.2 (3)	C21—C22—C23—C24	−84.2 (3)
C2—C3—C4—C6	−65.5 (3)	C21—C22—C23—C25	65.0 (3)
C2—C3—C4—C7	−133.7 (3)	C21—C22—C23—C26	133.1 (3)
C3—C2—N1—C1	7.6 (4)	C22—C21—N3—C20	−1.7 (4)
C3—C2—N1—C14	173.2 (2)	C22—C21—N3—C33	−169.5 (2)
C3—C4—C5—S1	−63.3 (3)	C22—C23—C24—S2	66.5 (3)
C3—C4—C6—C7	−106.7 (3)	C22—C23—C25—C26	106.9 (3)
C3—C4—C7—C6	107.9 (3)	C22—C23—C26—C25	−107.6 (3)
C4—C5—S1—C1	59.6 (3)	C23—C24—S2—C20	−60.9 (3)
C5—C4—C6—C7	104.2 (3)	C24—C23—C25—C26	−104.1 (3)
C5—C4—C7—C6	−109.6 (3)	C24—C23—C26—C25	109.0 (3)
C6—C4—C5—S1	86.2 (3)	C25—C23—C24—S2	−82.6 (3)
C7—C4—C5—S1	154.0 (2)	C26—C23—C24—S2	−150.1 (3)
C8—C1—N1—C2	−166.4 (2)	C27—C20—N3—C21	159.1 (2)
C8—C1—N1—C14	28.2 (3)	C27—C20—N3—C33	−33.2 (3)
C8—C1—S1—C5	153.1 (2)	C27—C20—S2—C24	−149.8 (2)
C8—C9—C10—C11	−0.7 (4)	C27—C28—C29—C30	−0.8 (5)
C9—C8—C13—C12	−0.6 (4)	C28—C27—C32—C31	1.2 (4)
C9—C10—C11—C12	−0.3 (4)	C28—C29—C30—C31	1.1 (5)
C10—C11—C12—C13	0.8 (4)	C29—C30—C31—C32	−0.2 (5)
C10—C11—C12—N2	−178.0 (2)	C29—C30—C31—N4	179.9 (3)
C11—C12—C13—C8	−0.4 (4)	C30—C31—C32—C27	−0.9 (4)
C11—C12—N2—O1	−6.4 (4)	C30—C31—N4—O4	−15.7 (4)
C11—C12—N2—O2	172.9 (3)	C30—C31—N4—O5	165.6 (3)
C13—C8—C9—C10	1.1 (4)	C32—C27—C28—C29	−0.4 (4)
C13—C12—N2—O1	174.8 (3)	C32—C31—N4—O4	164.4 (3)
C13—C12—N2—O2	−5.9 (4)	C32—C31—N4—O5	−14.4 (4)
C14—C15—C16—C17	0.0 (5)	C33—C34—C35—C36	−1.3 (5)
C15—C14—C19—C18	0.4 (4)	C34—C33—C38—C37	−0.2 (4)
C15—C14—N1—C1	−91.8 (3)	C34—C33—N3—C20	91.3 (3)
C15—C14—N1—C2	102.2 (3)	C34—C33—N3—C21	−100.4 (3)
C15—C16—C17—C18	0.3 (5)	C34—C35—C36—C37	0.2 (5)
C16—C17—C18—C19	−0.3 (5)	C35—C36—C37—C38	0.8 (5)
C17—C18—C19—C14	0.0 (5)	C36—C37—C38—C33	−0.9 (4)
C19—C14—C15—C16	−0.3 (4)	C38—C33—C34—C35	1.2 (4)
C19—C14—N1—C1	86.9 (3)	C38—C33—N3—C20	−86.4 (3)
C19—C14—N1—C2	−79.1 (3)	C38—C33—N3—C21	81.9 (3)
N1—C1—C8—C9	−87.2 (3)	N3—C20—C27—C28	−99.3 (3)
N1—C1—C8—C13	91.4 (3)	N3—C20—C27—C32	79.3 (3)
N1—C1—S1—C5	−83.0 (2)	N3—C20—S2—C24	84.2 (2)
N1—C2—C3—C4	−77.3 (3)	N3—C21—C22—C23	71.4 (3)
N1—C14—C15—C16	178.3 (3)	N3—C33—C34—C35	−176.5 (3)
N1—C14—C19—C18	−178.3 (3)	N3—C33—C38—C37	177.5 (2)
N2—C12—C13—C8	178.4 (2)	N4—C31—C32—C27	179.0 (2)
O3—C2—C3—C4	101.7 (3)	O6—C21—C22—C23	−106.5 (3)
O3—C2—N1—C1	−171.4 (2)	O6—C21—N3—C20	176.2 (2)
O3—C2—N1—C14	−5.8 (4)	O6—C21—N3—C33	8.4 (4)
S1—C1—C8—C9	37.6 (3)	S2—C20—C27—C28	134.0 (2)
S1—C1—C8—C13	−143.7 (2)	S2—C20—C27—C32	−47.4 (3)
S1—C1—N1—C2	70.4 (3)	S2—C20—N3—C21	−75.3 (3)
S1—C1—N1—C14	−94.9 (2)	S2—C20—N3—C33	92.4 (2)

### 6-(3-Nitrophenyl)-7-phenyl-5-thia-7-azaspiro[2.6]nonan-8-one (1) Hydrogen-bond geometry (Å, º)

**Table:**

*D*—H···*A*	*D*—H	H···*A*	*D*···*A*	*D*—H···*A*
C1—H1···O6	0.98	2.41	3.390 (3)	175
C3—H3*B*···O6	0.97	2.50	3.469 (3)	174
C10—H10···O6^i^	0.93	2.60	3.408 (3)	146
C17—H17···O2^ii^	0.93	2.46	3.216 (4)	138
C20—H20···O3^iii^	0.98	2.41	3.369 (3)	165
C28—H28···O3^iii^	0.93	2.56	3.384 (4)	147

Symmetry codes: (i) −*x*+1/2, *y*−1/2, −*z*+1/2; (ii) *x*, *y*−1, *z*; (iii) *x*, *y*+1, *z*.

### 6-(4-Nitrophenyl)-7-phenyl-5-thia-7-azaspiro[2.6]nonan-8-one (2) Crystal data

**Table:**

C_19_H_18_N_2_O_3_S	*D*_x_ = 1.332 Mg m^−^^3^
*M**_r_* = 354.41	Mo *K*α radiation, λ = 0.71073 Å
Orthorhombic, *P**c**a*2_1_	Cell parameters from 3246 reflections
*a* = 17.478 (3) Å	θ = 2.3–28.2°
*b* = 10.4125 (19) Å	µ = 0.20 mm^−^^1^
*c* = 9.7129 (17) Å	*T* = 298 K
*V* = 1767.6 (5) Å^3^	Needle, colorless
*Z* = 4	0.27 × 0.1 × 0.04 mm
*F*(000) = 744	

### 6-(4-Nitrophenyl)-7-phenyl-5-thia-7-azaspiro[2.6]nonan-8-one (2) Data collection

**Table:**

Bruker SMART CCD area detector diffractometer	4261 independent reflections
Radiation source: fine-focus sealed tube	2403 reflections with *I* > 2σ(*I*)
Parallel graphite monochromator	*R*_int_ = 0.056
Detector resolution: 8.34 pixels mm^-1^	θ_max_ = 28.3°, θ_min_ = 2.0°
phi and ω scans	*h* = −23→20
Absorption correction: multi-scan (SADABS; Bruker, 2001)	*k* = −13→13
*T*_min_ = 0.769, *T*_max_ = 0.9	*l* = −12→12
15240 measured reflections	

### 6-(4-Nitrophenyl)-7-phenyl-5-thia-7-azaspiro[2.6]nonan-8-one (2) Refinement

**Table:**

Refinement on *F*^2^	Secondary atom site location: difference Fourier map
Least-squares matrix: full	Hydrogen site location: inferred from neighbouring sites
*R*[*F*^2^ > 2σ(*F*^2^)] = 0.081	H-atom parameters constrained
*wR*(*F*^2^) = 0.249	*w* = 1/[σ^2^(*F*_o_^2^) + (0.1501*P*)^2^] where *P* = (*F*_o_^2^ + 2*F*_c_^2^)/3
*S* = 1.01	(Δ/σ)_max_ < 0.001
4261 reflections	Δρ_max_ = 0.55 e Å^−^^3^
226 parameters	Δρ_min_ = −0.30 e Å^−^^3^
1 restraint	Absolute structure: Flack (1983)
Primary atom site location: structure-invariant direct methods	Absolute structure parameter: 0.47 (19)

### 6-(4-Nitrophenyl)-7-phenyl-5-thia-7-azaspiro[2.6]nonan-8-one (2) Special details

**Table:**

Experimental. The data collection nominally covered a full sphere of reciprocal space by a combination of 4 sets of ω scans each set at different φ and/or 2θ angles and each scan (20 s exposure) covering -0.300° degrees in ω. The crystal to detector distance was 5.82 cm.
Geometry. All esds (except the esd in the dihedral angle between two l.s. planes) are estimated using the full covariance matrix. The cell esds are taken into account individually in the estimation of esds in distances, angles and torsion angles; correlations between esds in cell parameters are only used when they are defined by crystal symmetry. An approximate (isotropic) treatment of cell esds is used for estimating esds involving l.s. planes.
Refinement. Refinement of F^2^ against ALL reflections. The weighted R-factor wR and goodness of fit S are based on F^2^, conventional R-factors R are based on F, with F set to zero for negative F^2^. The threshold expression of F^2^ > 2sigma(F^2^) is used only for calculating R-factors(gt) etc. and is not relevant to the choice of reflections for refinement. R-factors based on F^2^ are statistically about twice as large as those based on F, and R- factors based on ALL data will be even larger.

### 6-(4-Nitrophenyl)-7-phenyl-5-thia-7-azaspiro[2.6]nonan-8-one (2) Fractional atomic coordinates and isotropic or equivalent isotropic displacement parameters (Å^2^)

**Table:**

	*x*	*y*	*z*	*U*_iso_*/*U*_eq_	
C1	0.6266 (3)	0.5630 (4)	0.3841 (4)	0.0635 (11)	
H1	0.6481	0.5871	0.4736	0.076*	
C2	0.7512 (2)	0.6299 (3)	0.2850 (5)	0.0559 (9)	
C3	0.7521 (3)	0.7400 (4)	0.3845 (4)	0.0593 (9)	
H3A	0.8027	0.7785	0.3838	0.071*	
H3B	0.7429	0.7071	0.4764	0.071*	
C4	0.6941 (3)	0.8423 (4)	0.3537 (5)	0.0684 (12)	
C5	0.6136 (3)	0.8248 (5)	0.4002 (8)	0.0895 (16)	
H5A	0.5854	0.9020	0.3769	0.107*	
H5B	0.6138	0.8180	0.4998	0.107*	
C6	0.5840 (3)	0.4389 (4)	0.4049 (5)	0.0677 (11)	
C7	0.6041 (3)	0.3639 (5)	0.5202 (6)	0.0840 (15)	
H7	0.6424	0.3920	0.5794	0.101*	
C8	0.5672 (4)	0.2488 (6)	0.5459 (8)	0.0912 (16)	
H8	0.5794	0.1990	0.6223	0.109*	
C9	0.5123 (3)	0.2113 (5)	0.4549 (7)	0.0836 (15)	
C10	0.4898 (3)	0.2793 (6)	0.3448 (8)	0.0952 (18)	
H10	0.4516	0.2491	0.2864	0.114*	
C11	0.5263 (3)	0.3988 (5)	0.3204 (7)	0.0794 (13)	
H11	0.5109	0.4499	0.2469	0.095*	
C12	0.6820 (2)	0.4565 (4)	0.1787 (4)	0.0550 (9)	
C13	0.7041 (3)	0.3314 (4)	0.1968 (6)	0.0697 (11)	
H13	0.7288	0.3067	0.2773	0.084*	
C14	0.6893 (3)	0.2408 (5)	0.0934 (6)	0.0780 (14)	
H14	0.7037	0.1555	0.1050	0.094*	
C15	0.6533 (3)	0.2795 (5)	−0.0250 (6)	0.0763 (13)	
H15	0.6425	0.2196	−0.0933	0.092*	
C16	0.6336 (3)	0.4032 (5)	−0.0435 (5)	0.0734 (12)	
H16	0.6105	0.4283	−0.1254	0.088*	
C17	0.6473 (3)	0.4935 (5)	0.0581 (5)	0.0646 (10)	
H17	0.6332	0.5787	0.0448	0.078*	
C18	0.7205 (4)	0.9800 (4)	0.3554 (6)	0.0846 (15)	
H18A	0.7735	0.9967	0.3789	0.102*	
H18B	0.6845	1.0446	0.3871	0.102*	
C19	0.7041 (4)	0.9149 (4)	0.2180 (5)	0.0822 (15)	
H19A	0.7474	0.8924	0.1603	0.099*	
H19B	0.6584	0.9404	0.1685	0.099*	
N1	0.69179 (18)	0.5453 (3)	0.2889 (3)	0.0568 (8)	
N2	0.4715 (4)	0.0877 (6)	0.4819 (10)	0.120 (2)	
O1	0.80230 (16)	0.6180 (3)	0.1992 (3)	0.0659 (7)	
O2	0.4223 (4)	0.0545 (6)	0.3992 (11)	0.169 (3)	
O3	0.4905 (4)	0.0269 (6)	0.5872 (9)	0.151 (3)	
S1	0.56211 (7)	0.69074 (13)	0.3337 (2)	0.0942 (5)	

### 6-(4-Nitrophenyl)-7-phenyl-5-thia-7-azaspiro[2.6]nonan-8-one (2) Atomic displacement parameters (Å^2^)

**Table:**

	*U*^11^	*U*^22^	*U*^33^	*U*^12^	*U*^13^	*U*^23^
C1	0.058 (2)	0.080 (3)	0.053 (2)	−0.0090 (19)	0.0124 (18)	−0.0078 (19)
C2	0.052 (2)	0.061 (2)	0.055 (2)	−0.0039 (17)	−0.0023 (19)	−0.0011 (18)
C3	0.064 (2)	0.063 (2)	0.050 (2)	−0.0029 (18)	−0.0001 (19)	−0.0036 (18)
C4	0.084 (3)	0.055 (2)	0.067 (3)	0.0001 (18)	−0.003 (2)	−0.005 (2)
C5	0.075 (3)	0.077 (3)	0.116 (4)	0.012 (2)	0.023 (3)	−0.008 (3)
C6	0.052 (2)	0.075 (3)	0.076 (3)	−0.0025 (19)	0.011 (2)	−0.002 (2)
C7	0.077 (3)	0.094 (4)	0.081 (4)	0.002 (3)	0.015 (3)	0.005 (3)
C8	0.086 (4)	0.091 (4)	0.096 (4)	0.007 (3)	0.017 (3)	0.010 (3)
C9	0.076 (3)	0.077 (3)	0.098 (4)	0.000 (2)	0.033 (3)	−0.005 (3)
C10	0.054 (3)	0.110 (4)	0.122 (5)	−0.014 (2)	0.015 (3)	−0.017 (4)
C11	0.059 (3)	0.088 (3)	0.091 (3)	−0.012 (2)	0.005 (3)	0.000 (3)
C12	0.053 (2)	0.064 (2)	0.049 (2)	−0.0016 (15)	−0.0009 (17)	−0.0045 (18)
C13	0.076 (3)	0.066 (2)	0.068 (3)	0.004 (2)	−0.007 (2)	0.004 (2)
C14	0.100 (4)	0.060 (3)	0.074 (3)	0.000 (2)	−0.001 (3)	−0.008 (2)
C15	0.079 (3)	0.077 (3)	0.073 (3)	−0.014 (2)	0.004 (3)	−0.015 (2)
C16	0.071 (3)	0.093 (3)	0.055 (2)	−0.015 (2)	−0.006 (2)	−0.006 (2)
C17	0.068 (3)	0.070 (3)	0.057 (2)	−0.006 (2)	−0.009 (2)	0.000 (2)
C18	0.099 (4)	0.061 (3)	0.095 (4)	−0.003 (2)	−0.014 (3)	−0.013 (3)
C19	0.112 (4)	0.074 (3)	0.060 (3)	−0.003 (3)	−0.020 (3)	0.005 (2)
N1	0.0504 (18)	0.072 (2)	0.0480 (18)	−0.0073 (13)	0.0019 (15)	−0.0070 (15)
N2	0.075 (3)	0.086 (3)	0.200 (7)	0.002 (3)	0.055 (4)	0.010 (5)
O1	0.0552 (16)	0.0804 (17)	0.0621 (18)	−0.0054 (13)	0.0086 (15)	−0.0031 (15)
O2	0.107 (4)	0.113 (4)	0.288 (10)	−0.043 (3)	0.024 (5)	−0.013 (5)
O3	0.148 (5)	0.105 (4)	0.201 (7)	−0.004 (3)	0.043 (5)	0.037 (4)
S1	0.0546 (6)	0.0848 (8)	0.1432 (14)	0.0082 (5)	−0.0026 (8)	−0.0113 (9)

### 6-(4-Nitrophenyl)-7-phenyl-5-thia-7-azaspiro[2.6]nonan-8-one (2) Geometric parameters (Å, º)

**Table:**

C1—H1	0.9800	C9—N2	1.494 (8)
C1—C6	1.505 (6)	C10—H10	0.9300
C1—N1	1.479 (5)	C10—C11	1.418 (8)
C1—S1	1.811 (5)	C11—H11	0.9300
C2—C3	1.500 (5)	C12—C13	1.370 (6)
C2—N1	1.361 (5)	C12—C17	1.373 (6)
C2—O1	1.228 (5)	C12—N1	1.425 (5)
C3—H3A	0.9700	C13—H13	0.9300
C3—H3B	0.9700	C13—C14	1.402 (7)
C3—C4	1.501 (6)	C14—H14	0.9300
C4—C5	1.488 (7)	C14—C15	1.371 (8)
C4—C18	1.506 (7)	C15—H15	0.9300
C4—C19	1.530 (7)	C15—C16	1.345 (8)
C5—H5A	0.9700	C16—H16	0.9300
C5—H5B	0.9700	C16—C17	1.384 (7)
C5—S1	1.782 (6)	C17—H17	0.9300
C6—C7	1.409 (8)	C18—H18A	0.9700
C6—C11	1.366 (7)	C18—H18B	0.9700
C7—H7	0.9300	C18—C19	1.524 (7)
C7—C8	1.384 (8)	C19—H19A	0.9700
C8—H8	0.9300	C19—H19B	0.9700
C8—C9	1.362 (9)	N2—O2	1.226 (11)
C9—C10	1.342 (9)	N2—O3	1.248 (10)
			
C6—C1—H1	106.9	C11—C10—H10	121.1
C6—C1—S1	111.0 (3)	C6—C11—C10	120.0 (6)
N1—C1—H1	106.9	C6—C11—H11	120.0
N1—C1—C6	111.0 (3)	C10—C11—H11	120.0
N1—C1—S1	113.7 (3)	C13—C12—C17	120.0 (4)
S1—C1—H1	106.9	C13—C12—N1	119.1 (4)
N1—C2—C3	119.0 (4)	C17—C12—N1	120.8 (4)
O1—C2—C3	120.4 (4)	C12—C13—H13	120.1
O1—C2—N1	120.6 (3)	C12—C13—C14	119.8 (5)
C2—C3—H3A	108.8	C14—C13—H13	120.1
C2—C3—H3B	108.8	C13—C14—H14	120.4
C2—C3—C4	114.0 (4)	C15—C14—C13	119.1 (5)
H3A—C3—H3B	107.6	C15—C14—H14	120.4
C4—C3—H3A	108.8	C14—C15—H15	119.6
C4—C3—H3B	108.8	C16—C15—C14	120.7 (5)
C3—C4—C18	117.8 (4)	C16—C15—H15	119.6
C3—C4—C19	116.4 (4)	C15—C16—H16	119.6
C5—C4—C3	119.4 (4)	C15—C16—C17	120.7 (5)
C5—C4—C18	113.8 (4)	C17—C16—H16	119.6
C5—C4—C19	115.5 (5)	C12—C17—C16	119.6 (4)
C18—C4—C19	60.2 (3)	C12—C17—H17	120.2
C4—C5—H5A	107.9	C16—C17—H17	120.2
C4—C5—H5B	107.9	C4—C18—H18A	117.7
C4—C5—S1	117.6 (4)	C4—C18—H18B	117.7
H5A—C5—H5B	107.2	C4—C18—C19	60.6 (3)
S1—C5—H5A	107.9	H18A—C18—H18B	114.8
S1—C5—H5B	107.9	C19—C18—H18A	117.7
C7—C6—C1	117.3 (5)	C19—C18—H18B	117.7
C11—C6—C1	123.2 (5)	C4—C19—H19A	117.9
C11—C6—C7	119.5 (5)	C4—C19—H19B	117.9
C6—C7—H7	119.8	C18—C19—C4	59.1 (3)
C8—C7—C6	120.5 (6)	C18—C19—H19A	117.9
C8—C7—H7	119.8	C18—C19—H19B	117.9
C7—C8—H8	121.3	H19A—C19—H19B	115.0
C9—C8—C7	117.4 (6)	C2—N1—C1	121.6 (3)
C9—C8—H8	121.3	C2—N1—C12	119.4 (3)
C8—C9—N2	118.0 (7)	C12—N1—C1	117.3 (3)
C10—C9—C8	124.9 (6)	O2—N2—C9	117.5 (8)
C10—C9—N2	117.1 (7)	O2—N2—O3	125.6 (7)
C9—C10—H10	121.1	O3—N2—C9	116.9 (8)
C9—C10—C11	117.7 (6)	C5—S1—C1	99.4 (2)
			
C1—C6—C7—C8	179.8 (5)	C12—C13—C14—C15	0.6 (8)
C1—C6—C11—C10	178.7 (5)	C13—C12—C17—C16	1.3 (7)
C2—C3—C4—C5	82.2 (6)	C13—C12—N1—C1	−92.4 (5)
C2—C3—C4—C18	−132.8 (4)	C13—C12—N1—C2	102.5 (5)
C2—C3—C4—C19	−64.1 (5)	C13—C14—C15—C16	1.2 (9)
C3—C2—N1—C1	3.0 (6)	C14—C15—C16—C17	−1.7 (8)
C3—C2—N1—C12	167.4 (4)	C15—C16—C17—C12	0.5 (7)
C3—C4—C5—S1	−61.4 (7)	C17—C12—C13—C14	−1.8 (7)
C3—C4—C18—C19	106.1 (5)	C17—C12—N1—C1	84.3 (5)
C3—C4—C19—C18	−108.4 (5)	C17—C12—N1—C2	−80.7 (5)
C4—C5—S1—C1	55.8 (5)	C18—C4—C5—S1	152.2 (4)
C5—C4—C18—C19	−106.9 (5)	C19—C4—C5—S1	85.2 (5)
C5—C4—C19—C18	104.0 (5)	N1—C1—C6—C7	95.0 (5)
C6—C1—N1—C2	−160.3 (4)	N1—C1—C6—C11	−87.0 (6)
C6—C1—N1—C12	35.0 (5)	N1—C1—S1—C5	−81.7 (4)
C6—C1—S1—C5	152.3 (4)	N1—C2—C3—C4	−71.9 (5)
C6—C7—C8—C9	0.9 (8)	N1—C12—C13—C14	175.0 (4)
C7—C6—C11—C10	−3.3 (8)	N1—C12—C17—C16	−175.4 (4)
C7—C8—C9—C10	−2.1 (9)	N2—C9—C10—C11	178.0 (5)
C7—C8—C9—N2	−179.5 (5)	O1—C2—C3—C4	106.4 (5)
C8—C9—C10—C11	0.5 (8)	O1—C2—N1—C1	−175.3 (4)
C8—C9—N2—O2	−179.8 (6)	O1—C2—N1—C12	−10.9 (6)
C8—C9—N2—O3	0.2 (8)	S1—C1—C6—C7	−137.6 (4)
C9—C10—C11—C6	2.3 (8)	S1—C1—C6—C11	40.5 (6)
C10—C9—N2—O2	2.5 (8)	S1—C1—N1—C2	73.7 (4)
C10—C9—N2—O3	−177.4 (6)	S1—C1—N1—C12	−91.0 (4)
C11—C6—C7—C8	1.7 (8)		

### 6-(4-Nitrophenyl)-7-phenyl-5-thia-7-azaspiro[2.6]nonan-8-one (2) Hydrogen-bond geometry (Å, º)

**Table:**

*D*—H···*A*	*D*—H	H···*A*	*D*···*A*	*D*—H···*A*
C1—H1···O1^i^	0.98	2.38	3.352 (6)	173
C3—H3*B*···O1^i^	0.97	2.48	3.444 (5)	171

Symmetry code: (i) −*x*+3/2, *y*, *z*+1/2.
